# *Orobanche crenata* Forssk. Extract Affects Human Breast Cancer Cell MCF-7 Survival and Viral Replication

**DOI:** 10.3390/cells11101696

**Published:** 2022-05-19

**Authors:** Carlo Genovese, Adriana Garozzo, Floriana D’Angeli, Giuseppe Antonio Malfa, Francesco Bellia, Barbara Tomasello, Daria Nicolosi, Roberta Malaguarnera, Simone Ronsisvalle, Fiorella Guadagni, Rosaria Acquaviva

**Affiliations:** 1 Faculty of Medicine and Surgery, “Kore” University of Enna, Contrada Santa Panasia, 94100 Enna, Italy; carlo.genovese@unikore.it (C.G.); roberta.malaguarnera@unikore.it (R.M.); 2 Nacture S.r.l., Spin-Off University of Catania, 95123 Catania, Italy; dnicolosi@unict.it (D.N.); s.ronsisvalle@unict.it (S.R.); racquavi@unict.it (R.A.); 3 Department of Biomedical and Biotechnological Sciences, Microbiology Section, University of Catania, 95123 Catania, Italy; agar@unict.it; 4 Department of Human Sciences and Quality of Life Promotion, San Raffaele Roma Open University, 00166 Rome, Italy; fiorella.guadagni@sanraffaele.it; 5 Department of Drug and Health Sciences, Biochemistry Section, University of Catania, 95125 Catania, Italy; g.malfa@unict.it (G.A.M.); btomase@unict.it (B.T.); 6 Research Centre on Nutraceuticals and Health Products (CERNUT), University of Catania, 95125 Catania, Italy; 7 Institute of Crystallography, National Research Council (CNR), 95126 Catania, Italy; francesco.bellia@cnr.it; 8 Department of Drug and Health Sciences, Microbiology Section, University of Catania, 95125 Catania, Italy; 9 Department of Drug and Health Sciences, Medicinal Chemistry Section, University of Catania, 95125 Catania, Italy; 10 BioBIM (InterInstitutional Multidisciplinary Biobank), IRCCS San Raffaele Pisana, 00166 Rome, Italy

**Keywords:** *Orobanche crenata* extract, MCF-7 cells, MDA-MB-231 cells, *Herpes simplex* virus, *Coxsackievirus*, parasitic plant

## Abstract

Background: Breast cancer (BC) is the leading cause of death worldwide. The severity of BC strictly depends on the molecular subtype. The less aggressive hormone-positive subtype is treated with adjuvant endocrine therapy (AET), which causes both physical and psychological side effects. This condition strongly impacts the adherence and persistence of AET among oncologic patients. Moreover, viral infections also constitute a serious problem for public health. Despite their efficacy, antiviral agents present several therapeutic limits. Accordingly, in the present work, we investigated the antitumor and antiviral activities of *Orobanche crenata* Forssk. (*O. crenata*), a parasitic plant, endemic to the Mediterranean basin, traditionally known for its beneficial properties for human health. Methods: The MTT assay was carried out to evaluate the cytotoxic effect of *O. crenata* leaf extract (OCLE) on human breast cancer cells (MCF-7 and MDA-MB-231) and the primary HFF-1 cell line. The lactic dehydrogenase (LDH) assay was performed on MCF-7 cells to analyze necrotic cell death. The antioxidant effect of OCLE was evaluated by intracellular determination of the reactive oxygen species and thiol groups, by DPPH and ABTS assays. The antiviral activity of OCLE was determined against *Poliovirus* 1, *Echovirus* 9, Human respiratory syncytial virus, *Adenovirus* type 2 and type 5, *Coxsackievirus* B1 (CoxB1) and B3 (CoxB3), *Herpes simplex* type 1 (HSV-1) and type 2 (HSV-2), and β-*Coronavirus* by the plaque reduction assay. Results: The extract, after 24 h of incubation, did not affect MDA-MB-231 and HFF-1 cell viability. However, at the same time point, it showed a dose-dependent inhibitory effect on MCF-7 cells, with an increase in LDH release. OCLE exhibited free radical scavenging activity and significantly increased non-protein thiol levels in MCF-7 cells. OCLE effectively inhibited HSV-1, HSV-2, CoxB1, and CoxB3 replication. Conclusions: The overall results showed an interesting inhibitory effect of OCLE on both MCF-7 cell survival and viral replication.

## 1. Introduction

Breast cancer (BC) represents one of the most common female cancers. A recent report estimated 287,850 expected new cases of BC and 43,250 related deaths, in 2022, in the United States [1]. The high incidence of BC reflects the enormous clinical and economical efforts for public health in the management of this chronic disease [2,3]. Progress in BC treatment has allowed the quality of life of oncologic patients to be improved, their life expectancy extended, and ultimately, reducing the mortality rate [4]. The survival of patients strictly depends on the molecular features of breast cancer cells [5]. Clinically, it is possible to distinguish different subtypes of BC, depending on the expression of estrogen receptor (ER), progesterone receptor (PR), and human epidermal growth factor receptor (HER-2) [6]. Hormone receptor-positive BC represents the majority of BCs [7]. These subtypes are associated with a more favorable prognosis because they are highly responsive to adjuvant endocrine therapy (AET). This consists of the use of selective estrogen receptor modulators, such as tamoxifen and raloxifene, aromatase inhibitors, and GnRH agonists, which efficiently counteract the tumorigenic effect of estradiol [8]. Despite the efficacy, AET induces both physical and psychological side effects, which influence patients’ compliance, particularly in pre-menopausal women [9,10]. Indeed, a large portion (ranging from 30% to 70%) of breast cancer survivors do not comply with the prescribed doses and frequency of AET (adherence) or assume it discontinuously (persistence) [11]. This phenomenon negatively impacts survival and the risk of recurrence of the disease [12]. In light of this, research on potential adjuvants of natural origin that affect, in a targeted manner, breast cancer cell survival could represent a useful approach to eliminate or mitigate the side effects of AET, improving the adherence of oncologic patients to the therapy.

A further threat to public health is constituted by viral infections. The pandemic caused by the SARS-Cov-2 virus highlighted the high vulnerability of individuals to these microorganisms. A large variety of viruses, with different organ tropism, endanger the health of millions of individuals every year, worldwide. In this regard, *Poliovirus* (Polio), *Coxsackievirus* (Cox), *Echovirus* (ECHO) [13], *Adenovirus* (Adeno) [14], Respiratory syncytial virus (RSV) [15], *Herpes simplex* virus 1 (HSV-1) and 2 (HSV-2) [16], and β-*Coronavirus*, such as human coronavirus OC-43 [17], are only some examples of viral agents able to cause a wide spectrum of diseases of variable severity in humans. The treatment of virus-related infections is often complicated by different factors, including the scarce availability of antiviral drugs, especially in the case of emerging new viruses, the toxicity of viral antimicrobials to host organisms, and the ability of these microorganisms to develop resistance against the antivirals [18]. Accordingly, there is an urgent need for new and effective antiviral agents that could help to control viral infections.

The anticancer and antimicrobial effects of natural extracts [19,20,21,22,23,24,25,26] are well documented. The biological activities of phytoextracts are mediated by a mix of molecules that singularly or in combination can damage cancer cells and pathogens, including viruses [19,27,28]. The biological properties of the natural extracts are tightly correlated with the plants from which they are derived. One of these, the parasitic plant *Orobanche crenata* Forssk. (*O. crenata*), distinguishes itself for its interesting antimicrobial properties. *O. crenata* is a species belonging to the Orobanchaceae family, order Lamiales, found mainly in temperate regions of the Mediterranean basin and western Asia through to Iran [29]. It is an annual chlorophyll-free herbaceous parasitic of leguminous plants, with high-persistence seeds that are viable for over 10 years, which when stimulated by specific mediators of the leguminous root system, emit a rootlike structure (haustorium) and penetrate the host’s root to drain the lymph [30]. Its shoot consists of alternate leaves reduced to somewhat fleshy sessile scales, and a crenate earing that opens into a white-purple inflorescence [31]. The immature shoots are used to prepare dishes that are traditionally consumed in the rural communities of the Mediterranean basin [32], whereas the flowering ones are also used in folk medicine due to their beneficial properties for human health [30].

In our previous paper, we demonstrated the antimicrobial activity of *O. crenata* leaf extract (OCLE) on several clinically important bacterial and fungal strains [33]. Moreover, in a further study, we proved the antifungal action of OCLE on two *Candida* strains responsible for severe ocular infections: *Candida albicans* (*C. albicans*) and *Candida glabrata*. Specifically, we showed the ability of OCLE to counteract the growth and adhesion of the two strains to host cells. Moreover, the extract was also able to inhibit the phenotypic switching of *C. albicans* and promote the repair of the retinal epithelium. These activities were correlated with the presence of different classes of secondary metabolites, such as phenylpropanoid glycosides, phenolic aldehydes, and flavones, detected, in the extract, by ultra-performance liquid chromatography-tandem mass spectrometry (UPLC-Ms/Ms) [34].

Concerning the antitumor activity, a recent study highlighted the cytotoxic effect of the methanolic extract obtained from the entire *O. crenata* plant on different cancer cell lines, including MCF-7 cells. However, in this work, the authors examined in depth the antitumor effect of the extract on the human colon carcinoma cell line HCT-116 [35].

Accordingly, the promising biological activities of *O. crenata* extract led us to extend the analysis of OCLE by exploring its effect on human breast cancer cell lines (MCF-7, MDA-MB-231) and several clinically relevant viruses.

## 2. Materials and Methods

### 2.1. Chemical Reagents

All chemicals were purchased from Sigma-Aldrich (Taufkirchen; Germany), except those mentioned elsewhere.

### 2.2. Preparation of OCLE Extract

**Figure 1:**
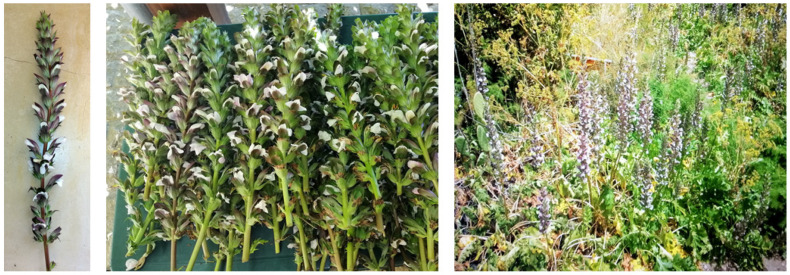
*Orobanche crenata* Forssk.

*O. crenata* (Figure 1) was collected in Modica (Ragusa, Italy, latitude 36°51048.9300 N, longitude 14°45053.6900 E, altitude 382 m) in May 2019, and a voucher specimen (n. 35/04) was deposited in the herbarium of the Department of Drug and Health Sciences, University of Catania. OCLE was obtained as reported in our previous work [33].

### 2.3. Antitumor Activity

#### 2.3.1. Cell Culture

The human breast cancer cell line MCF-7 (ATCC^®^ HTB-22™; ATCC, Manassas, VA, USA) and human breast cancer cell line MDA-MB-231 (ATCC^®^ HTB-26™; ATCC, Manassas, VA, USA) were cultured in RPMI medium containing 10% *v*/*v* FBS, 100 U/mL penicillin, and 100 mg/mL streptomycin, at 37 °C with a 5% CO_2_ humidified incubator. The human foreskin fibroblast cell line HFF-1 (ATCC^®^ SCRC-1041 ™; ATCC, Manassas, VA, USA) was maintained in DMEM (Sigma-Aldrich s.r.l., Milan, Italy) supplemented with 15% *v*/*v* FBS, 4.5 g/L glucose, 100 U/mL penicillin, and 100 mg/mL streptomycin, in 95% humidified air with 5% CO_2_, at 37 °C. HFF-1 was used as in vitro human model for preliminary toxicity screening. For testing, MCF-7, MDA-MB-231, and HFF-1 cells were seeded in 96-well microplates at a constant density (8 × 10^3^ cells/well) and experiments were started after 24 h [21].

#### 2.3.2. MTT Assay

After 24 h of incubation in a humidified atmosphere of 5% CO_2_ at 37 °C and under sub-confluent conditions, MCF-7, MDA-MB-231, and HFF-1 cells were treated with different concentrations of OCLE (from 75 to 1200 µg/mL) for 24 h.

The assay measures the conversion of tetrazolium salt to yield colored formazan in the presence of metabolic activity. The amount of formazan is proportional to the number of living cells. The absorbance of the converted formazan was measured using a microplate spectrophotometer reader (Titertek Multiskan, Flow Laboratories, Helsinki, Finland) at *λ* = 570 nm. The results were presented as the percent of control data [21].

#### 2.3.3. Lactic Dehydrogenase Release

Lactic dehydrogenase (LDH) release was measured to evaluate necrotic cell death because of cell membrane disruption. LDH activity was measured spectrophotometrically in the culture medium and the cellular lysates, at *λ* = 340 nm, by analyzing nicotinamide adenine dinucleotide (NAD) reduction [21]. The percentage of LDH release was calculated as the percentage of the total amount, considered as the sum of the enzymatic activity present in the cellular lysate and that in the culture medium. Results were expressed as the percentage of LDH released.

#### 2.3.4. Intracellular Reactive Oxygen Species Assay

MCF-7 cells (density of 8 × 10^3^ cells/well) were incubated with the extract dilutions (from 75 to 1200 μg/mL) for 24 h. The 2,7-dichlorodihydrofluorescein diacetate (DCFH-DA) solution was added to each well and the 96-well microplate was incubated at 37 °C. The assay was performed to quantify the reactive oxygen species (ROS) levels as previously described [36]. The intensity of dichlorofluorescein fluorescence was measured by fluorescence spectrophotometry (excitation, *λ* = 488 nm; emission, *λ* = 525 nm). Results were expressed as fluorescence intensity/mg protein and, for each sample, the total protein content was determined using the Sinergy HTBiotech instrument by measuring the absorbance difference at *λ* = 280 nm and *λ* = 260 nm.

#### 2.3.5. Thiol Group Determination

MCF-7 cells were plated in 12-well plates (3 × 10^5^ cells/well) and incubated at 37 °C in a humidified atmosphere with 5% CO_2_. After 24 h, the cells were treated with different concentrations of extract (from 75 to 1200 µg/mL) for 24 h. Total non-protein thiol groups (GSH) were determined spectrophotometrically at *λ* = 412 nm using 5,5′-dithiobis (2-nitrobenzoic acid) [36]. Results were expressed as nmol GSH/mg proteins, which was calculated by referring to a glutathione calibration curve. For each sample, the total protein content was determined as described for the ROS assay.

### 2.4. Antioxidant Activity

#### 2.4.1. DPPH Assay

The 2,2-diphenyl-1-picrylhydrazyl (DPPH) radical was used to test the scavenger activity of OCLE. We used a method previously reported with some modifications to adapt the protocol to a microplate reader (Varioskan Flash, Thermo Scientific, Waltham, MA, USA) [37]. Briefly, DPPH was dissolved in ethanol (5 mM) and properly diluted to obtain an optical density (O.D.) value at *λ* = 517 nm lower than 1.0. The final DPPH solution was mixed to different concentrations of the extract, obtained through a 2-fold dilution series starting from 400 µg/mL. The absorbance at *λ* = 517 nm was read after 10 min, at room temperature, in the dark. The DPPH inhibition was calculated as follows:

$$\mathrm{DPPH}\mathrm{inhibition}=\frac{{\mathrm{Abs}}_{\mathrm{control}}-{\mathrm{Abs}}_{\mathrm{sample}}}{{\mathrm{Abs}}_{\mathrm{control}}}\times 100$$

These data were fitted to the following equation:

$$y=\frac{a}{1+e^{-\frac{x-{\mathrm{IC}}_{50}}{b}}}$$

where IC_50_ is the OCLE concentration that reduces 50% of the absorbance due to DPPH.

#### 2.4.2. ABTS Assay

The decoloration assay of the 2,2′-azinobis(ethylbenzothiazoline-6-sulphonic acid) radical cation (ABTS) was adapted for a microplate reader (Varioskan Flash, Thermo Scientific, Waltham, MA, USA) [37]. ABTS (7 mM) and potassium persulphate (2.5 mM) were dissolved in water and kept overnight at room temperature in the dark. The final solution was properly diluted to obtain an absorbance value close to 0.7 at *λ* = 734 nm. OCLE and 6-hydroxy-2,5,7,8-tetramethylchroman-2-carboxylic acid (Trolox) (0–600 μM) were diluted in phosphate buffer (1 mM, pH 7.4) along with the ABTS solution. The absorbance at *λ* = 734 nm was monitored after 10 min of reaction. Trolox was used as a standard and it was assayed (0–100 µM) as reported for OCLE. The activity of the latter was reported as Trolox equivalents, i.e., the standard concentration that produces an absorbance variation equal to that of the tested compound. Three independent experiments were carried out, and the mean data with standard deviations were reported.

### 2.5. Antiviral Activity

#### 2.5.1. Cell Viability

The cytotoxicity of OCLE on human epidermoid carcinoma larynx cells (HEp-2; ATCC^®^ CCL-23™; ATCC, Manassas, VA, USA), African green monkey kidney cells (Vero; ATCC^®^ CCL-81™; ATCC, Manassas, VA, USA), and Cellosaurus HCT-8 (HCT-8; ATCC^®^ CCL-244™; ATCC, Manassas, VA, USA) cell lines was determined by measuring the effect on the morphology and growth. Cell monolayers were prepared in 96-well tissue culture microplates (Corning) and exposed to different concentrations of the natural extract. The microplates were checked by light microscopy after 24, 48, and 72 h. The cytotoxicity was scored as morphological alterations (e.g., rounding up, shrinking, detachment). The viability assay was carried out through the 3-[4,5-dimethylthiazol-2-yl]-2,5 diphenyl tetrazolium bromide (MTT)-based In Vitro Toxicology Assay Kit (product number TOX1-1KT, Merck, Germany) after 48 and 72 h of exposure. Briefly, the cells were seeded at a density of 2 × 10^5^ cells/mL in 96-well tissue culture microplates, such that the cell replication remained logarithmic for the incubation time. Each vial of MTT (product number M-5655, Sigma-Aldrich, Milan, Italy) was reconstituted with 3 mL of the appropriate medium without phenol red and serum. In total, 10 µL of reconstituted MTT were added to the culture medium (10% *v*/*v*). After 3 h of incubation, 100 µL of MTT Solubilization Solution (product number M-8910, Sigma-Aldrich, Milan, Italy) were added to the microplates to dissolve the resulting formazan crystals. The O.D. was read at *λ* = 540 and *λ* = 690 nm by a microplate reader (Gen5 Microplate Reader, BioTek Instruments, Winooski, VT, USA). The absorbance at *λ* = 690 nm was automatically subtracted from the absorbance at *λ* = 540 nm to eliminate the effect of non-specific absorption. The 50% cytotoxic dose (CD_50_) was defined as the highest concentration of the substance that resulted in a 50% cell growth reduction compared with the control cultures [19,38].

#### 2.5.2. Antiviral Activity of OCLE

All the viruses were purchased from the American Type Culture Collection (ATCC^®^). *Poliovirus* 1 (Polio 1: ATCC^®^ VR-1000^TM^, Brunhilde strain), *Echovirus* 9 (ECHO 9: ATCC^®^ VR-39^TM^, Hill strain), Human respiratory syncytial virus (RSV: ATCC^®^ VR-2454^TM^), and *Adenovirus* type 2 (Adeno 2: ATCC^®^ VR-1080^TM^) and type 5 (Adeno 5: ATCC^®^ VR-1516^TM^) were propagated in HEp-2 cells at 37 °C. *Coxsackievirus* B1 (COX B1: ATCC^®^ VR-687^TM^), *Coxsackievirus* B3 (COX B3: ATCC^®^ VR-30^TM^), and *Herpes simplex* type 1 (HSV-1: ATCC^®^ VR-260^TM^) and type 2 (HSV-2: ATCC^®^ VR-734^TM^) were propagated in Vero cells at 37 °C. β-*Coronavirus* (OC-43: ATCC^®^ VR-1558^TM^) was propagated in HCT-8 cells at 33 °C. HEp2 and Vero cells were kept in a humidified 5% carbon dioxide atmosphere at 37 °C and grown in Dulbecco modified Eagle’s Minimum Essential Medium (DMEM) supplemented with 6% *v*/*v* heat-inactivated fetal calf serum (FCS). HCT-8 cells were grown using Roswell Park Memorial Institute-1640 (RPMI-1640) medium supplemented with 10% *v*/*v* heat-inactivated FCS. All the culture media were supplemented with 200 µg/mL of streptomycin and 200 units/mL of penicillin G (Gibco^TM^). For all the viruses, tested working stocks were prepared as cellular lysates using the medium with 2% *v*/*v* FCS (maintenance medium) [19,38]. The antiviral effect of OCLE was evaluated following different schemes of treatment, based on the tested virus species. The standard drug acyclovir was used as the positive control.

#### 2.5.3. Plaque Reduction Assay

The antiviral activity of OCLE was assessed by the 50% plaque reduction assay. Confluent cells were grown in 96-well tissue culture microplates and infected with the viral suspension. During and after 1 h of virus adsorption at 37 °C (30 min for Picornaviruses), an overlay medium containing 1% *v*/*v* of methylcellulose with or without the test compound at doses below CD_50_ was added. After 24–48 h of incubation at 37 °C, when the plaques appeared clearly in virus controls, the overlay was removed, and cells were stained with 1% *v*/*v* crystal violet in methanol. The number of visible plaques was then counted under light microscopy. The antiviral activity of each compound was determined as the percentage decrease in the number of plaques. The compound concentration required to inhibit virus plaque formation by 50% was expressed as ID_50_ [19,38].

#### 2.5.4. Cytopathic Effect Inhibition Assays

The infectivity of OC-43 stock was determined by the MTT method: the reciprocals of viral dilution that resulted in a 50% reduction in the absorbance of formazan in the infected cells at 48–72 h was determined as the infectivity of the virus by MTT ID_50_ (50% infective dose). The anti-*Coronavirus* assay was based on the inhibition of virus-induced cytopathogenicity on HCT-8 cells. Briefly, sub-confluent monolayers grown in 96-well tissue culture microplates were treated with or without different concentrations of the test compound at concentrations below the CD_50_, and then infected with 10 CDID_50_ (50% cell culture infective dose) of the virus stock to produce a complete cytopathic effect within 72–96 h. After incubation at 33 °C, the viability of mock-infected and virus-infected cells was quantified by the MTT method. The compound concentration required to inhibit the virus-induced cytopathogenicity by 50% was expressed as ID_50_ [19,38].

### 2.6. Chemical Profile of OCLE

The phytochemical profile of OCLE was determined by UPLC-Ms/Ms, as reported in our previous work [34].

### 2.7. Statistical Analysis

One-way analysis of variance (ANOVA) was performed to estimate significant differences among the treatments. All the results were obtained by three independent experiments each performed in triplicate (i.e., biological and technical triplicates). Data analysis and graphical representations were performed by using GraphPad Prism 8 software (GraphPad, San Diego, CA, USA).

## 3. Results

### 3.1. Antitumor Activity of OCLE

#### 3.1.1. MTT Assay

**Figure 2:**
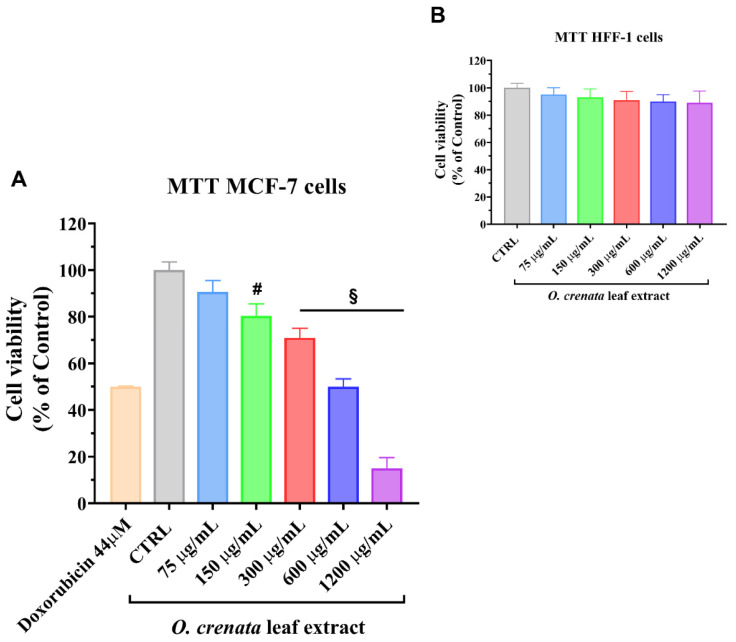
Cell viability of MCF-7 cells (**A**) and HFF-1 cells (**B**) untreated (control; CTRL) and treated for 24 h with increasing concentrations (from 75 to 1200 μg/mL) of *O. crenata* leaf extract. Experiments were performed using Doxorubicin as a standard cytotoxic compound. The IC_50_ of the standard agent was 44 ± 0.3 μM for MCF-7 cells and >100 μM for HFF-1 cells (not shown). Values are the mean ± SD of four experiments in triplicate. § *p* < 0.0001, # *p* < 0.001 vs. untreated control.

OCLE did not affect HFF-1 cell viability at all the tested concentrations after 24 h of exposure (Figure 2A). Conversely, at the same time point, the treatment with increasing concentrations of OCLE, from 75 to 1200 μg/mL, induced a dose-dependent inhibitory effect on the succinate dehydrogenase activity of the MCF-7 cells, with an EC_50_ value of 396.6 μg/mL (Figure 2A). The OCLE extract did not induce any change in cell viability on MDA-MB-231 breast cancer cells (metastatic, basal, triple-negative) at the tested concentration range (Figure S1).

#### 3.1.2. Lactic Dehydrogenase Release

**Figure 3:**
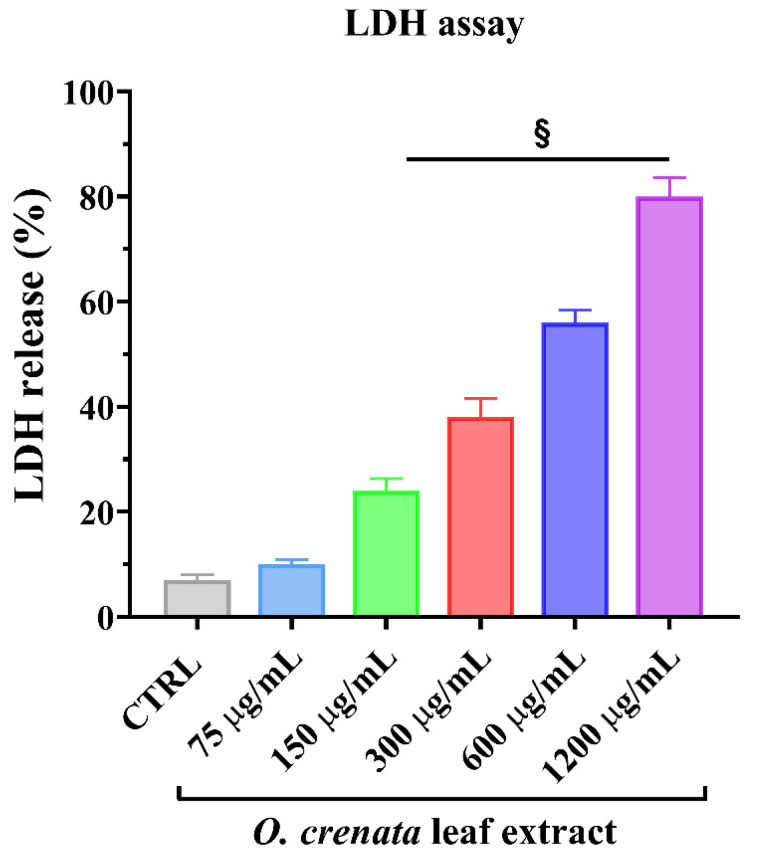
Lactic dehydrogenase (LDH) is released from MCF-7 cells at the steady state (control, CTRL) and after 24 h of treatment with increasing concentrations (from 75 to 1200 μg/mL) of OCLE. Values are the mean ± SD of four experiments in triplicate. § *p* < 0.0001 vs. untreated control.

As shown in Figure 3, the presence of the natural extract at different concentrations in the culture medium determined a significant dose-dependent increase in LDH release from MCF-7 cells. At the concentration of 600 µg/mL, the LDH release was about 50%. The obtained results in the LDH assay confirmed that most of the antiproliferative activity of OCLE, observed by the MTT assay, is associated with necrotic cellular death.

### 3.2. Antioxidant Activity of OCLE

#### 3.2.1. Reactive Oxygen Species Levels

**Figure 4:**
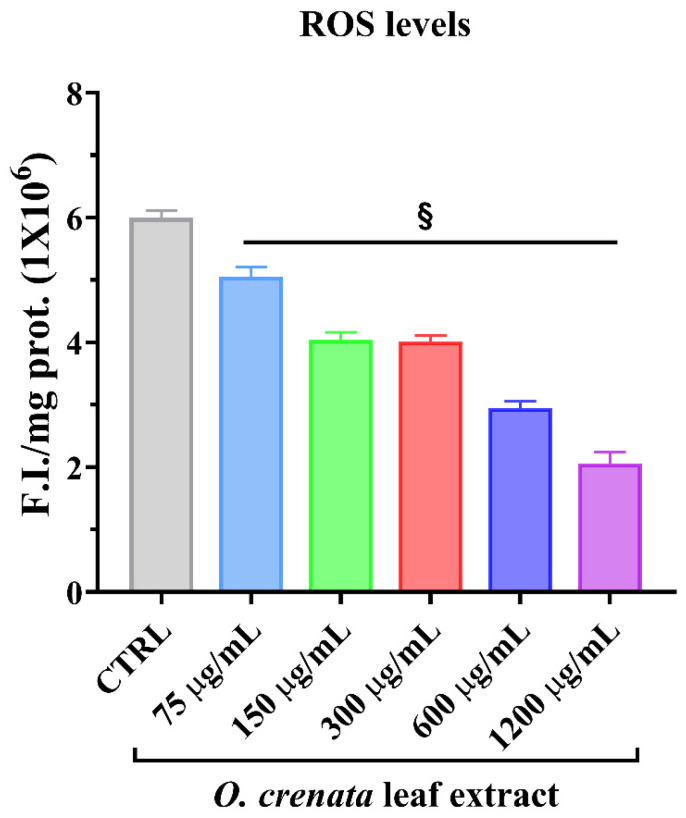
Intracellular oxidants in MCF-7 cells untreated and treated for 24 h with different concentrations (75–1200 μg/mL) of *O. crenata* leaf extract. Values are the mean ± SD of four experiments in triplicate. § *p* < 0.0001 vs. untreated control (CTRL).

It is well known that ROS levels are significantly raised in tumor cells because of different factors. Indeed, increases in ROS promote tumor progression. Figure 4 shows that the treatments of MCF-7 cells with different concentrations of OCLE resulted in a significant decrease in radical species already at the lowest concentration tested (75 μg/mL).

#### 3.2.2. Thiol Group Determination

**Figure 5:**
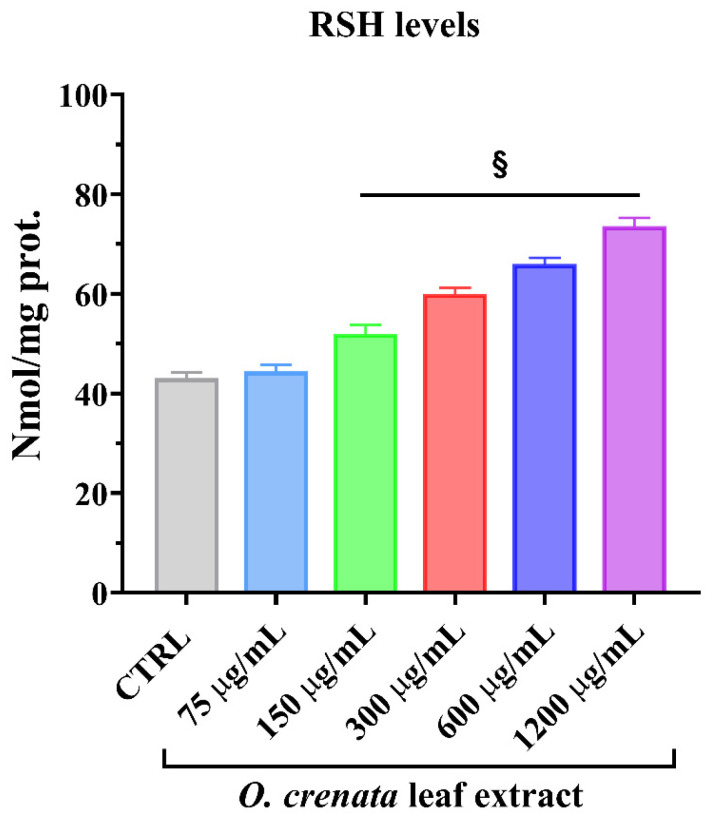
Thiol groups in MCF-7 cells untreated and treated for 24 h with different concentrations (75–1200 μg/mL) of *O. crenata* leaf extract. Values are the mean ± SD of four experiments in triplicate. § *p* < 0.0001 vs. untreated control (CTRL).

ROS can react with different cellular substrates. Specifically, intracellular thiol residues are one of the main targets. Besides, thiols play a key role in cancer cell survival and growth. The tested concentrations of OCLE significantly increased non-protein thiol levels in MCF-7 cells with respect to the untreated cells (Figure 5). The reported increase in the thiols amount seems to be strictly associated with the decrease in ROS levels (Figure 4).

#### 3.2.3. DPPH and ABTS Assays

**Figure 6:**
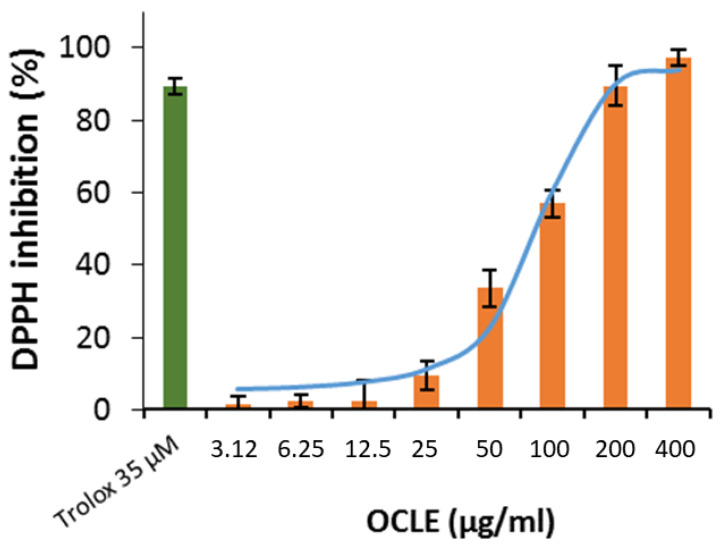
Scavenger activity against DPPH of *O. crenata* leaf extract (OCLE). The results are expressed as a percentage of the decrease in absorbance at λ = 517 nm compared to the standard Trolox. Each value represents the mean ± SD of three experimental measurements.

The antioxidant activity of OCLE was determined by two different stable radicals: DPPH and ABTS. Figure 6 clearly shows that the scavenger activity of OCLE against DPPH increases in a dose-dependent manner. The fitted IC_50_ linked to the sigmoidal trend is 81.3 µM, meaning that the extract contains several compounds (flavonoid and anthocyanins, among others) that can counteract free-radical species.

**Figure 7:**
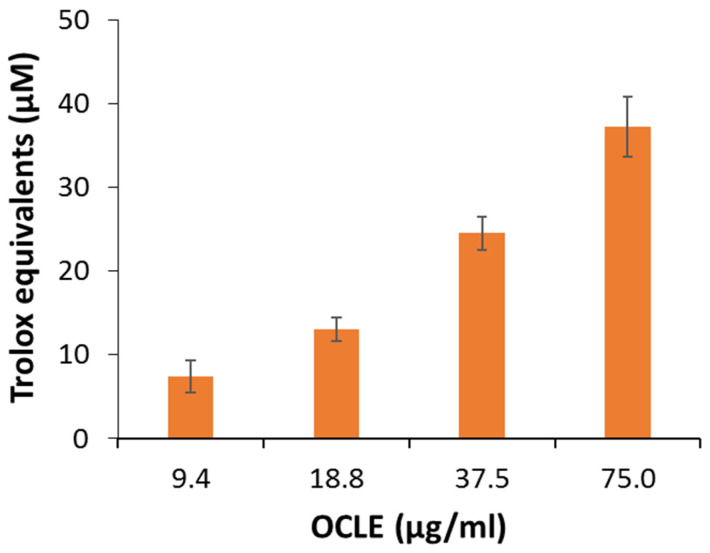
Scavenger activity against ABTS of *O. crenata* leaf extract (OCLE). The results are expressed as Trolox equivalents. Each value represents the mean ± SD of three experimental measurements.

The capacity of OCLE to quench free-radical species was also confirmed when ABTS was used to test the scavenger activity. Once again (Figure 7), OCLE exerted antioxidant activity in a dose-dependent manner. The activity was equivalent to quite low amounts of Trolox, a water-soluble vitamin E analogue, which was used as a standard. This confirmed that the antioxidant power of the extract should be able to protect cellular environments against oxidative stress.

### 3.3. Antiviral Activity of OCLE

#### Cell Viability

Table 1 reports the CD_50_ values of OCLE on the Vero, HEp-2, and HCT-8 cell lines and the ID_50_ values against Polio 1, Cox B1, Cox B3, ECHO 9, RSV, Adeno 2, Adeno 5, HSV-1, HSV-2, and OC43. OCLE showed a CD_50_ value of 600 µg/mL for the Vero and HEp-2 cell lines and 300 µg/mL for the HCT-8 cell line. Concerning the antiviral effect of OCLE, it exhibited better activity against HSV-1, with an ID_50_ value of 50 µg/mL. For HSV-2 and Cox B1, an IC_50_ value of 100 µg/mL was obtained. However, weak activity was observed for Cox B3 (200 µg/mL). OCLE was found to be inactive against the remaining viruses tested.

### 3.4. Chemical Profile of OCLE

UPLC-Ms/Ms-detected molecules and their mass spectra are shown in Table 2 and Figures S2–S13, respectively. The chemical analysis of OCLE was performed in our previous work [34]. Accordingly, here, we report selected molecules endowed with antiviral, antitumor, and antioxidant activities. A careful analysis of literature data revealed a strong activity of the compounds luteolin and salidroside. Therefore, the notable biological activities found in this work could be due to these two molecules.

## 4. Discussion

It is well known that chemotherapy significantly compromises the quality of life of oncologic patients [75]. Regarding hormone-positive BC, the efficiency of AET is counterposed to the onset of side effects, which are sometimes severe enough to affect the adherence and persistence of breast cancer survivors to therapy, abolishing its health-promoting effects [76]. In this scenario, an integrative treatment with natural adjuvants, endowed with tumor-specific anticancer activity, could be considered a good strategy to limit the adverse effects induced by chemotherapy, thus promoting the adherence of oncologic patients to the recommended treatment regimen.

Traditionally, medicinal plants were widely used to alleviate or treat chronic and infectious diseases [77]. Through the extraction process, it is possible to obtain the biologically active molecules contained in the plants. However, the chemical composition and, consequently, the biological activity of the natural extracts depends on a series of factors, including the selected plant; the geographic area in which it is grown; the extraction conditions, such as solvent/s, temperature, pressure, and time; and finally, in the case of parasitic plants, the host plant [78,79,80,81,82]. In the last decades, the investigation of the biological activities mediated by phytoextracts has sparked great interest. Specifically, in the oncological field, the toxicity induced by chemotherapeutic drugs makes the research and development of new, specific, and well-tolerated anticancer agents necessary [83]. The parasitic plant *O. crenata* is an edible plant that typically grows in Mediterranean areas [30]. The extracts of the different parts of *O. crenata* showed interesting pharmacological activities, including antioxidant [33,84], anti-inflammatory [85], antimicrobial [33,34,84], and anticancer activities [35].

Given the outstanding biological effects of *O. crenata* extracts, in the present study, we proposed an analysis of the antitumoral action of the *O. crenata* leaf extract (OCLE) against the ER-positive human breast cancer cell line MCF-7 and triple-negative MDA-MB-231 breast cancer cells, by exposing these cells to different concentrations of the extract (from 75 to 1200 μg/mL) for 24 h. The MTT assay showed a significant reduction in MCF-7 cell viability, already at 150 μg/mL. Our results are consistent with those obtained by Hegazy et al., who demonstrated the cytotoxic effect of methanolic extract of *O. crenata* on different human cell lines, including MCF-7 cells. Specifically, the authors tested increasing concentrations of the extract, ranging from 3.9 to 500 μg/mL, obtaining a dose-dependent reduction in MCF-7 cell viability [35]. However, the MTT assay and the microscopic analysis of MCF-7 cells revealed a strong toxic effect of the extract at 500 μg/mL, whereas, in our case, a similar effect was obtained at the highest tested concentration (Figure 2). This discrepancy can be attributed to a series of factors that influenced the extraction process, as mentioned above. Indeed, in that study, the extract was obtained from the whole plant, which was grown in Egypt. Moreover, the solvent, temperature, and time used for the extraction were different from that used in our study [35]. Taken together, these factors are responsible for the different chemical compositions of the two extracts, which reflects the different biological activities [86].

The cytotoxic effect of the extract on MCF-7 cells was further analyzed to establish whether the extract was able to induce uncontrolled cell death of human breast cancer cells. For this purpose, we evaluated the lactic dehydrogenase (LDH) release from MCF-7 after the treatment with increasing concentrations of the extract (Figure 3). Interestingly, OCLE determined a significant and dose-dependent increase in LHD release in MCF-7 cells compared to the control (untreated cells). It has been shown that treatment of the human colorectal cancer cell line HT29 with salidroside, an O-glycosyl compound previously detected in OCLE (Table 2) by UPLC-Ms/Ms [34], induced a significant increase in cell death markers, such as LDH and cleaved caspase-3 [87]. The antitumoral effect of such molecules was also proved on several cancer cell lines [88,89,90,91], including MCF-7 cells. An interesting study demonstrated the inhibitory effect of salidroside on the proliferation of both ER-negative (MDA-MB-231 cells) and ER-positive (MCF-7 cells) breast cancer cells. Moreover, this molecule stimulated apoptotic cell death in both cell lines, highlighting its promising role as an antineoplastic agent for breast cancer treatment [70]. However, although our extract contains this compound and others with proven anticancer activity against MDA-MB-231 breast cancer cells, in our case, we found that OCLE did not affect the viability of this cell line at all the tested concentrations (Figure S1). This phenomenon could be due to a combination of factors related to cells and the extract. Specifically, the triple-negative MDA-MB-231 cells are characterized by higher resistance to the treatment compared to ER-positive MCF-7 cells [92,93]. Furthermore, as previously mentioned, the extract contains a pool of bioactive compounds, which are present at low concentrations. On the other hand, literature data reported a potent anti-breast cancer activity of pure compounds, including salidroside (see Table 2). Accordingly, the differences in the anticancer activity between the extract and the pure molecules lie in the possibility to test the activity of the latter at higher concentrations compared to those present in the extract, thus enhancing their intrinsic biologic effect [94,95,96].

Further evidence of the anti-breast cancer activity of salidroside derives from an in vitro and in vivo study, which confirmed the anti-proliferative and pro-apoptotic action of such molecule on MCF-7 cells and showed an ability to counteract tumor growth in a nude mouse model. In addition, in that study, the authors demonstrated that salidroside inhibited intracellular ROS production in MCF-7 cells in a dose-dependent manner [68]. It is well known that ROS play a crucial role in tumor progression and invasion [97,98]. Indeed, the active metabolism of transformed cells causes an increment in ROS levels [99]. In a tumoral context, the oxidative cell damage induced by ROS is responsible for genetic instability, which, in turn, can lead to the loss of function of genes involved in the control of cell growth, thus promoting the proliferation and invasion of cancer cells [93,94,95]. In this regard, in our study, we demonstrated a significant and dose-dependent reduction in intracellular ROS levels in MCF-7 cells treated with OCLE (Figure 4). By contrasting the increment in ROS levels, OCLE showed a potential antineoplastic action against human breast cancer cells. Nevertheless, excess ROS production can provoke irreversible cell damage, which culminates in oxidative apoptosis [100,101]. To prevent this fatal event, cells have evolved different antioxidant systems and molecules, through which they neutralize the toxic effects of oxygen. Among such components, the intracellular non-protein thiol groups, of which glutathione (GSH) is the most representative, assume a relevant role as one of the main non-enzymatic mechanisms involved in the maintenance of the redox balance [99,102]. Regarding cancer cell survival, GSH has a dual function due to its antioxidant role. Indeed, in the presence of elevated levels of ROS, GSH plays a protective role by counteracting oxidative cell damage. During this activity, GSH is converted to the oxidate form glutathione disulfide (GSSG). Accordingly, depletion of the GSH reserves exacerbates the deleterious effects of ROS, triggering apoptotic cell death. On the other hand, GSH is involved in the detoxification of cells from protumor agents, and limitations in GSH availability make cells more susceptible to the tumor-promoting action of ROS [103,104]. Therefore, to ensure their survival, cancer cells must maintain a delicate equilibrium between oxidant and antioxidant production. In our case, the treatment of MCF-7 cells with OCLE induced a significant increment in non-protein thiols (Figure 5) as a direct consequence of the inhibitory activity of the extract against ROS. The reduction in the intracellular ROS levels and the concomitant increase in GSH availability proved the ability of the extract to modulate the redox status of breast cancer cells, hindering the carcinogenic effect of oxidative stress. Further proof of the antioxidant action of OCLE is given by its scavenger activity against the two radicals DPPH (Figure 6) and ABTS (Figure 7). This capacity could be due to the presence, in the extract, of several compounds with proven antioxidant activity (Table 2). Specifically, the previously performed UPLC-Ms/Ms [34] detected the presence of the triterpenoid acteoside [41,43], the flavones apigenin [45,46,105,106,107,108,109,110] and luteolin [57,61,62,63,64,111,112,113,114], and the O-glycosyl compound salidroside [68,69,70,71,72,73,74] in OCLE, which showed both antioxidant activity and anticancer effects against MCF-7 cells. Therefore, extensive literature data strongly support our findings, which could be the result of a synergistic interaction between these active molecules.

Besides the limits of cancer therapy, the current period is highlighting the difficulty in treating viral infections. The scarce availability of efficient antiviral agents makes individuals extremely susceptible to viruses. Considering that in our previous works we demonstrated notable antibacterial and antifungal activities of OCLE [33,34] and that the antiviral properties of *O. crenata* extract have not yet been explored, in the present study, we focused on this biological activity by evaluating the effect of OCLE on several medically important viruses. Accordingly, in our experimental investigations, we found that the extract exerted an antiviral effect against HSV (HSV-1 and HSV2) and COX (B1 and B3 strains). It is worth highlighting that acteoside, a phenylpropanoid glycoside derived from different plant species, can prevent HSV-1 adsorption and inhibit HSV-2 attachment and penetration [40]. The flavonoid luteolin acts in the early steps of COX B3 replication by reducing the viral yield and suppressing the synthesis of RNA and proteins [115]. Interestingly, in an in silico analysis, luteolin also showed an antiviral potential against the SARS-CoV-2 virus through the inhibition of RNA replication and viral reactivation, respectively [53]. Furthermore, Wang et al. demonstrated the protective effect of salidroside on myocardial cells against COX B3 infections, probably due to the modulation of antioxidant enzymes and genes related to apoptosis [65]. Based on literature evidence, the different compounds contained in the extract could act in synergy by affecting virus replication stages or by interacting with specific viral glycoproteins [116].

## 5. Conclusions

In the present study, we found that OCLE significantly affects human breast cancer cell survival by inducing necrotic cell death and regulating the oxidative status of cancer cells. Moreover, the extract showed interesting antiviral effects, mainly against HSV-1, HSV-2, COX B1, and COX B3. The chemical analysis revealed the presence of bioactive molecules that could be responsible for the observed effects. Therefore, our study adds another piece to the puzzle of the biological activities of *O. crenata* extract, highlighting its potential as both a chemotherapeutic and antiviral co-adjuvant. Considering this, we propose that the understanding of such aspects is deepened in the future by isolating one or more chemical compounds (e.g., apigenin) present in the extract to investigate its/their effects on MCF-7 cells and the tested viruses and the molecular mechanisms underlying the anticancer and antiviral activities.

## Figures and Tables

**Table 1:** Antiviral activity of OCLE and acyclovir.

		OCLE ^a^ (µg/mL)	Acyclovir (µg/mL)
**CD_50_ ^b^**	Vero	600	OR ^c^
	HEp-2	600	OR
	HCT-8	300	OR
**ID_50_ ^d^**	Polio 1	>600	OR
	Cox B1	100	OR
	Cox B3	200	OR
	ECHO 9	>600	OR
	RSV	>600	OR
	Adeno 2	>600	OR
	Adeno 5	>600	OR
	HSV-1	50	0.12
	HSV-2	100	0.36
	OC-43	>300	OR

^a^ OCLE: *Orobanche crenata* leaf extract; ^b^ CD_50_ represents the concentration that inhibited 50% cell growth compared to the control; ^c^ OR: out of range of concentration; ^d^ ID_50_ represents the concentration that inhibited 50% virus plaque formation and virus-induced cytopathogenicity. Values are mean ± 0.5 S.D. (maximal S.D. estimated) for 3 separate assays.

**Table 2:** Biological activities (antiviral, antitumor on the MCF-7 cell line, and antioxidant) of the chemical compounds identified in *O. crenata* leaf extract by UPLC-Ms/Ms.

Chemical Name	Chemical Class	Chemical Structure	*m*/*z* (g/mol)	Polarity	Peak	RT (min)	Biological Activities
Acteoside	Phenylpropanoid glycosides	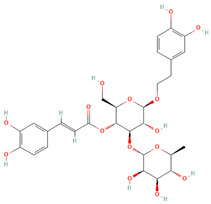	624.205	Pos	625.21	23.23	Antiviral [39,40] Antitumor (MCF-7) [41,42] Antioxidant [43]
				Neg	623.19		
Apigenin	Flavones	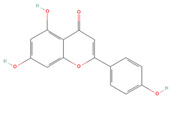	270.053	Pos	271.06	35.97	Antiviral [44] Antitumor (MCF-7) [45] Antioxidant [46]
				Neg	269.04		
Acutissimin A	Complex tannins	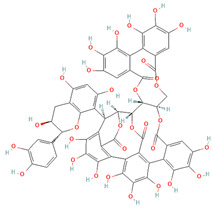	1206.822	Pos	1207.14	n.r.	Antiviral [47] Antioxidant [48]
				Neg	1205.10		
Crenatoside	Phenylpropanoid glycosides	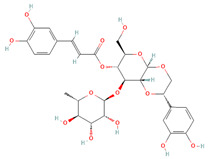	622.190	Pos	n.r.	54.02	Antiviral [49] Antioxidant [50]
				Neg	n.r.		
Luteolin	Flavones	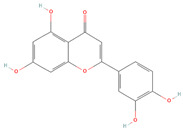	286.048	Pos	287.05	21.85	Antiviral [51,52,53,54] Antitumor (MCF-7) [55,56,57,58,59,60] Antioxidant [61,62,63,64]
				Neg	285.03		
Salidroside	O-glycosyl compounds	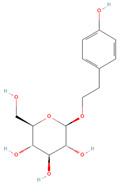	300.121	Pos	301.12	35.10	Antiviral [65,66,67] Antitumor (MCF-7) [68,69,70] Antioxidant [71,72,73,74]
				Neg	299.10		

Notes: RT: retention time; n.r.: not reported, due to the low concentration of the compound in the extract.

## Data Availability

The data presented in this study are available on request from the corresponding author.

## Supplementary Materials

The following supporting information can be downloaded at: www.mdpi.com/article/10.3390/cells11101696/s1, Figure S1: Cell viability of MDA-MB-231 cells untreated (control; CTRL) and treated for 24 h with increasing concentrations (from 75 to 1200 μg/mL) of *O. crenata* leaf extract. Experiments were performed using Doxorubicin as the standard cytotoxic compound. The IC50 of the standard agent was 50 ± 1.1μM. Values are the mean ± SD of four experiments in triplicate; Figures S2–S7: Mass spectrum of *Orobanche crenata* leaf extract (positive polarity—Q1 mode); Figures S8–S13: Mass spectrum of *Orobanche crenata* leaf extract (negative polarity—Q1 mode).
