# Supplementary material for: Orobanche crenata Forssk. Extract Affects Human Breast Cancer Cell MCF-7 Survival and Viral Replication
Source: Cells. 2022 May 19;11(10):1696. doi: 10.3390/cells11101696 (PMC9139723; doi:10.3390/cells11101696)
Supplement: Supplementary file 1 [file cells-11-01696-s001.zip › cells-1699694-supplementary.pdf]

cells

Supplementary Materials

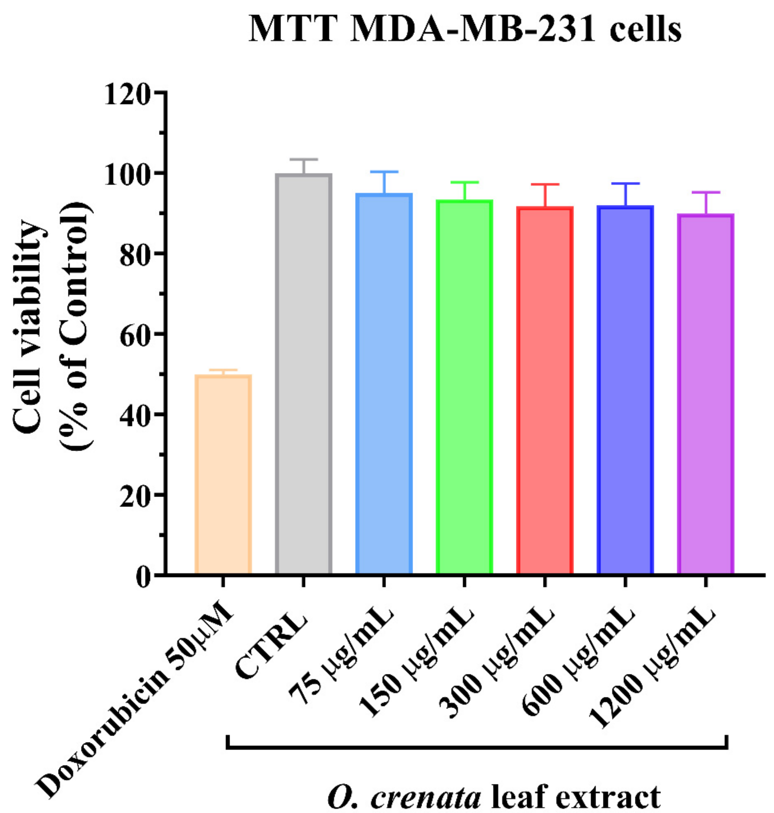

Figure S1. Cell viability of MDA-MB-231 cells untreated (control; CTRL) and treated for 24 h with increasing concentrations (from 75 to 1200 µg/mL) of *O. crenata* leaf extract. Experiments were performed using Doxorubicin as standard cytotoxic compound. The IC50 of the standard agent was 50±1.1µM. Values are the mean ± SD of four experiments in triplicate.

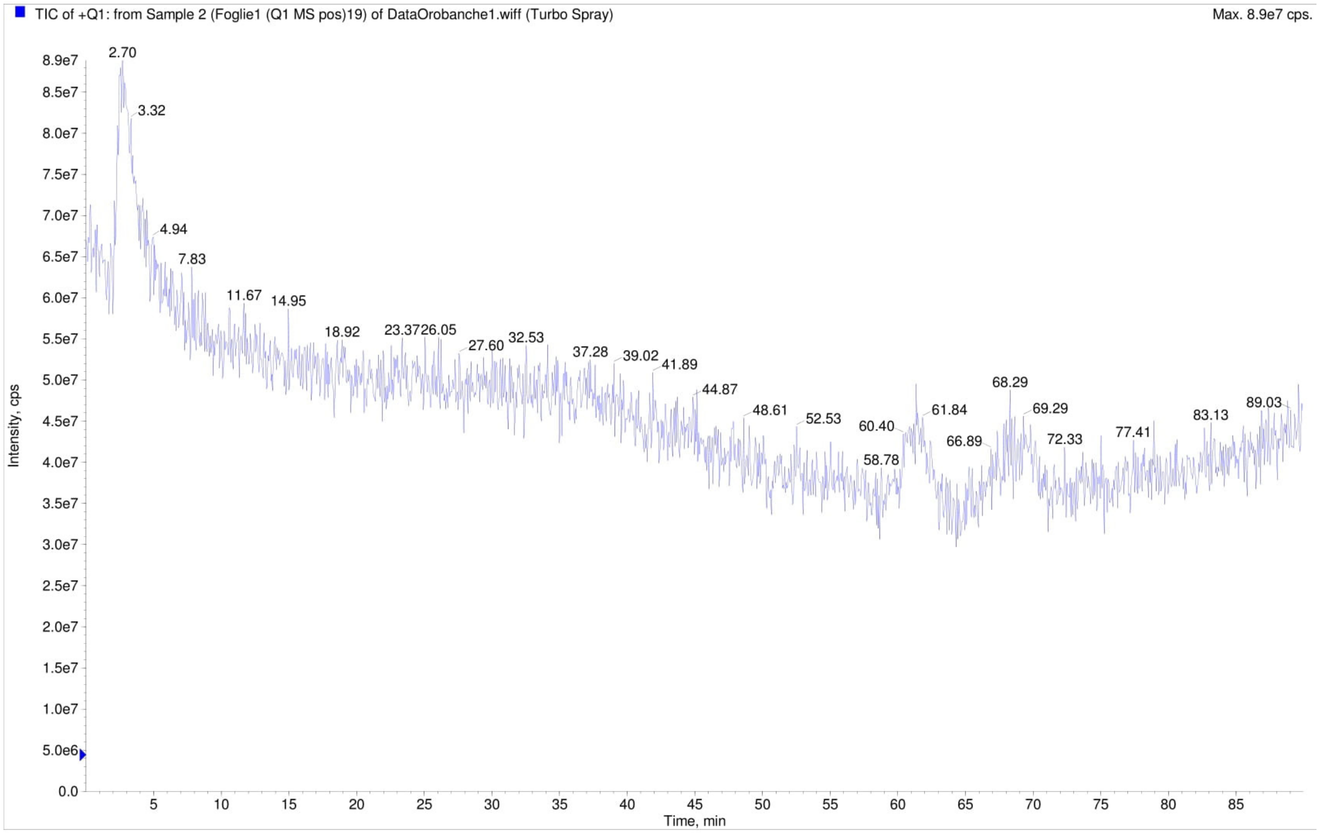

Figure S2. Mass spectrum of *Orobanche crenata* leaf extract (positive polarity – Q1 mode). In x-axis is reported the retention time (min).

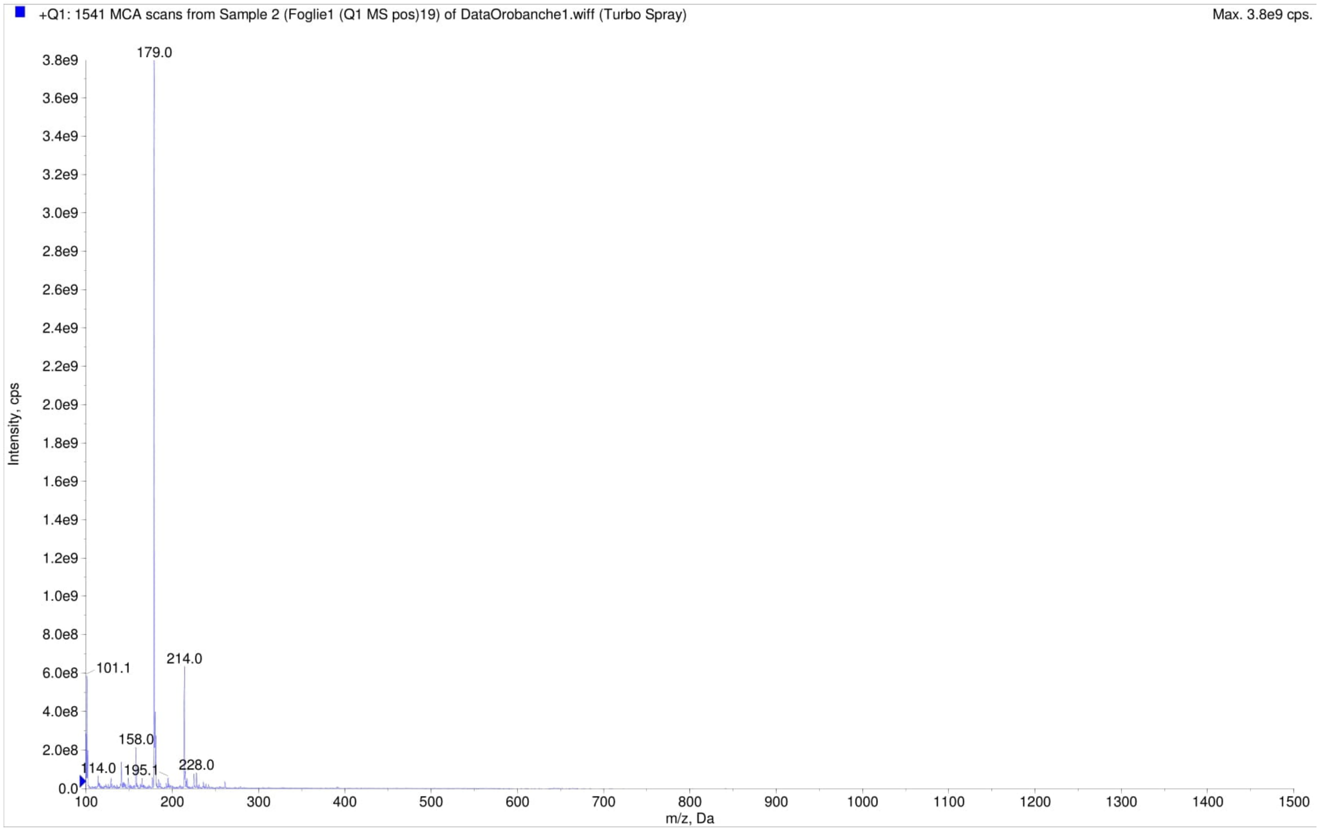

Figure S3. Mass spectrum of *Orobanche crenata* leaf extract (positive polarity – Q1 mode). In x-axis is reported the mass-to-charge ratio (m/z) from 100 to 1500 Da.

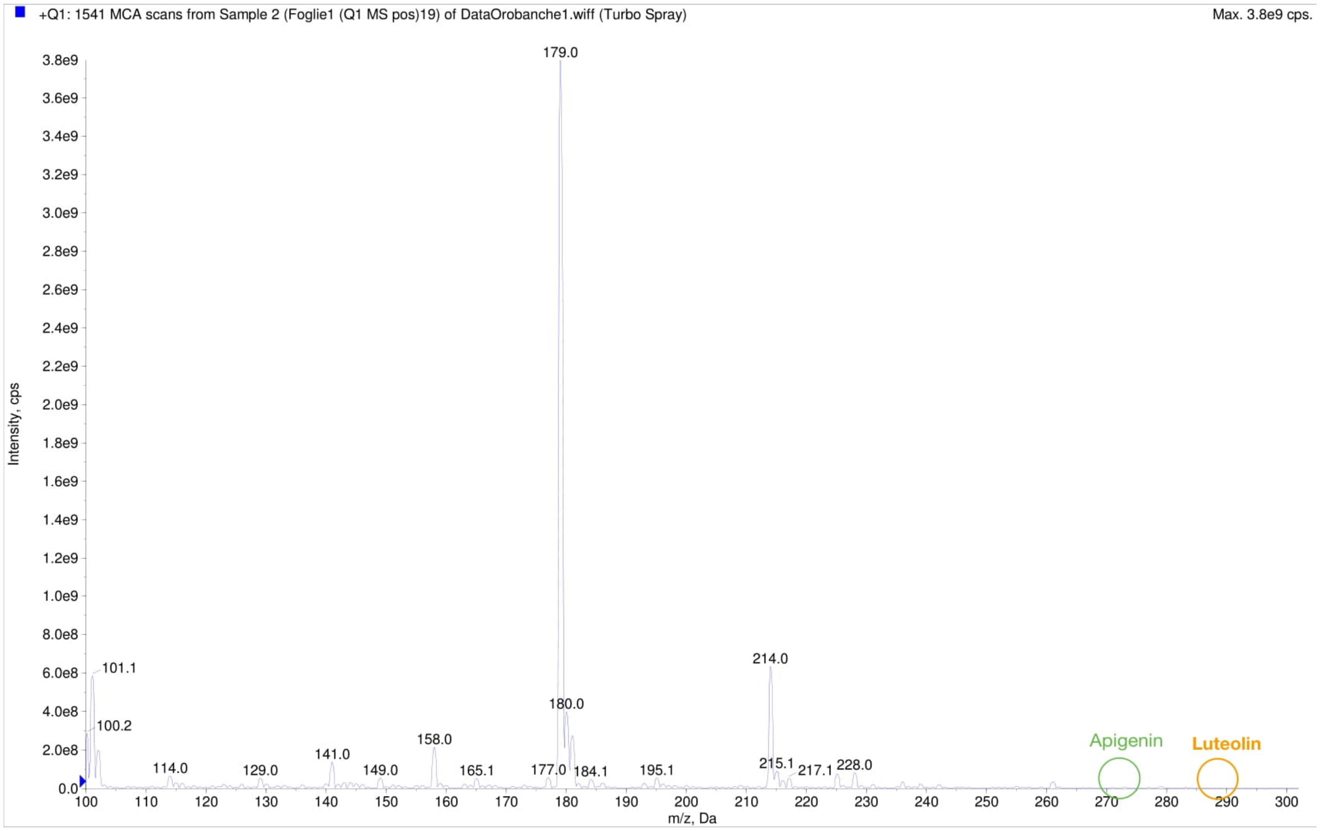

Figure S4. Mass spectrum of *Orobanche crenata* leaf extract (positive polarity – Q1 mode). In x-axis is reported the mass-to-charge ratio (m/z) from 100 to 300 Da.

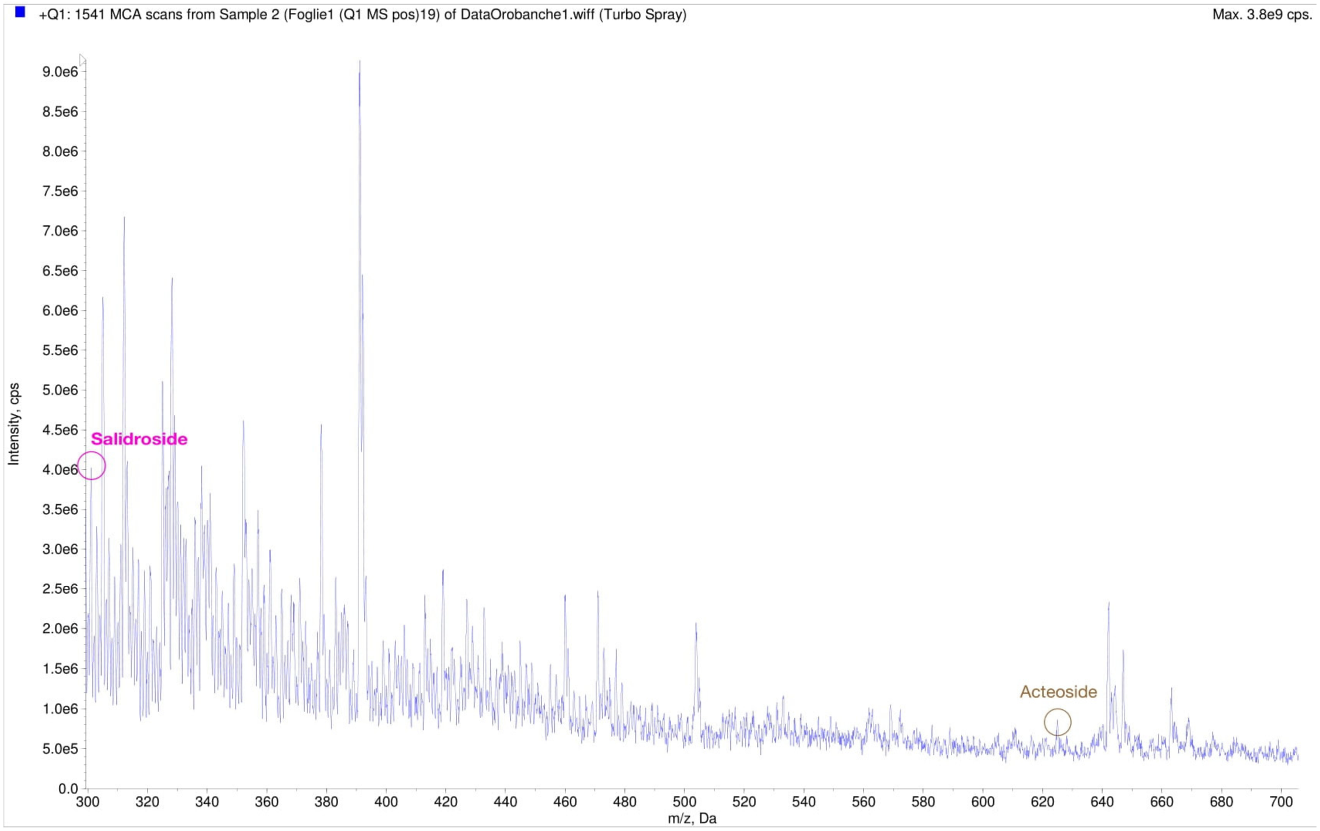

Figure S5. Mass spectrum of *Orobanche crenata* leaf extract (positive polarity – Q1 mode). In x-axis is reported the mass-to-charge ratio (m/z) from 300 to 700 Da.

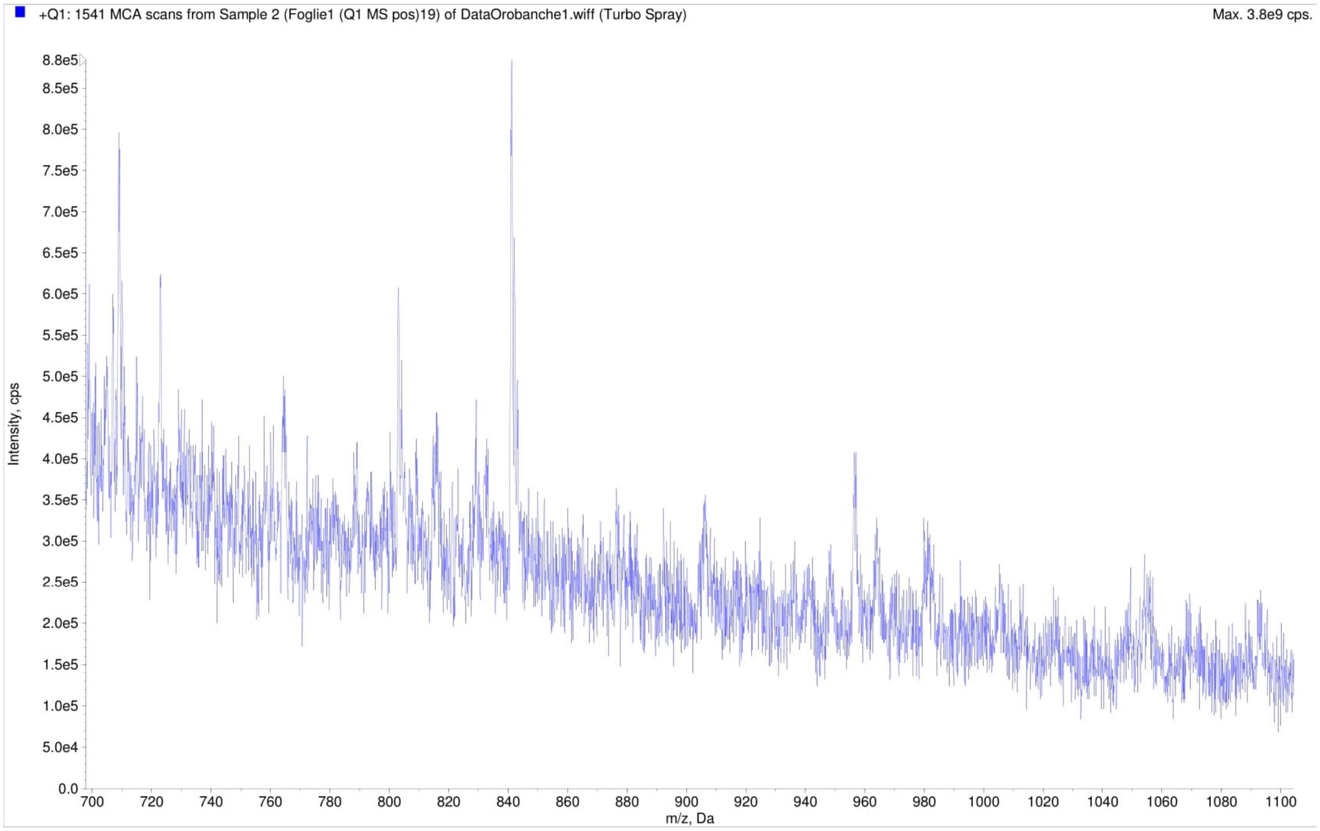

Figure S6. Mass spectrum of *Orobanche crenata* leaf extract (positive polarity – Q1 mode). In x-axis is reported the mass-to-charge ratio (m/z) from 700 to 1100 Da.

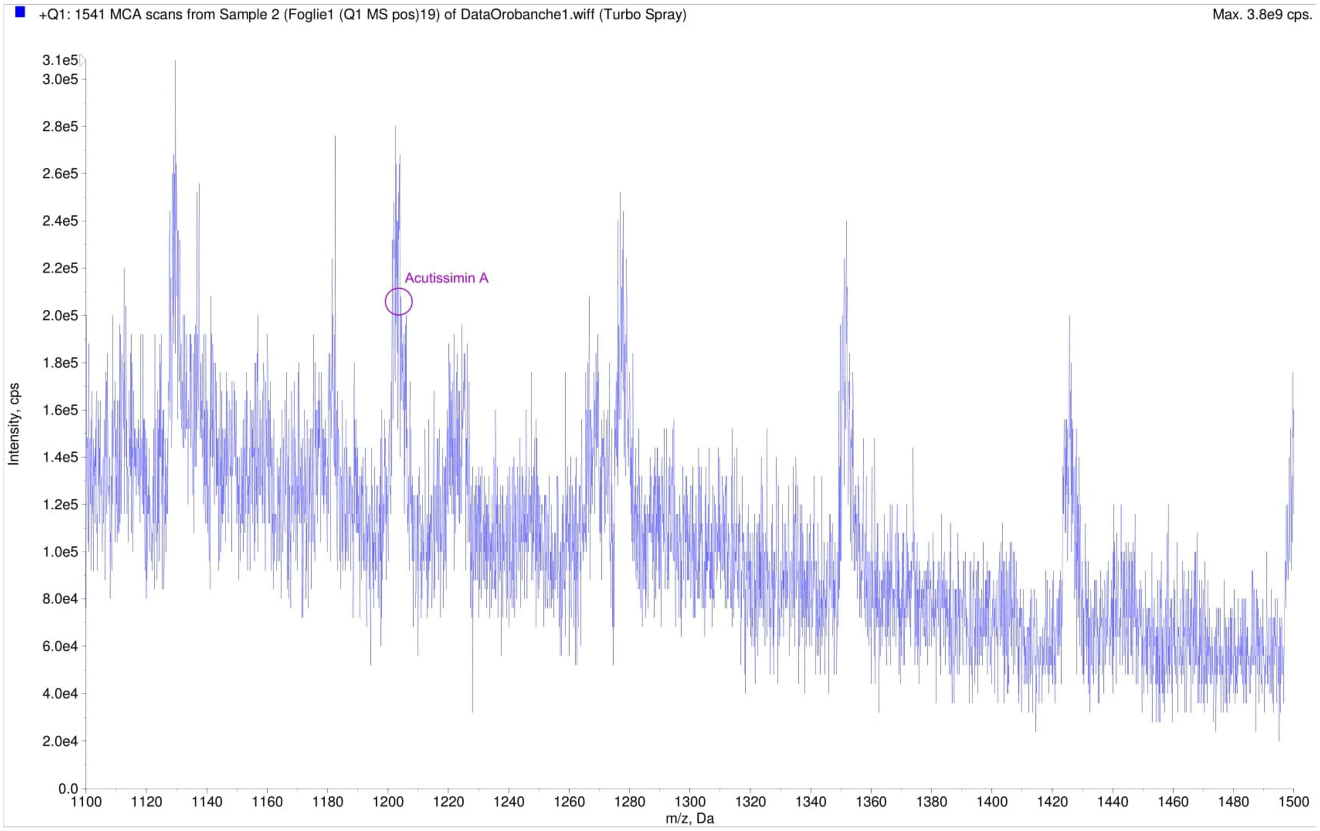

Figure S7. Mass spectrum of *Orobanche crenata* leaf extract (positive polarity – Q1 mode). In x-axis is reported the mass-to-charge ratio (m/z) from 1100 to 1500 Da.

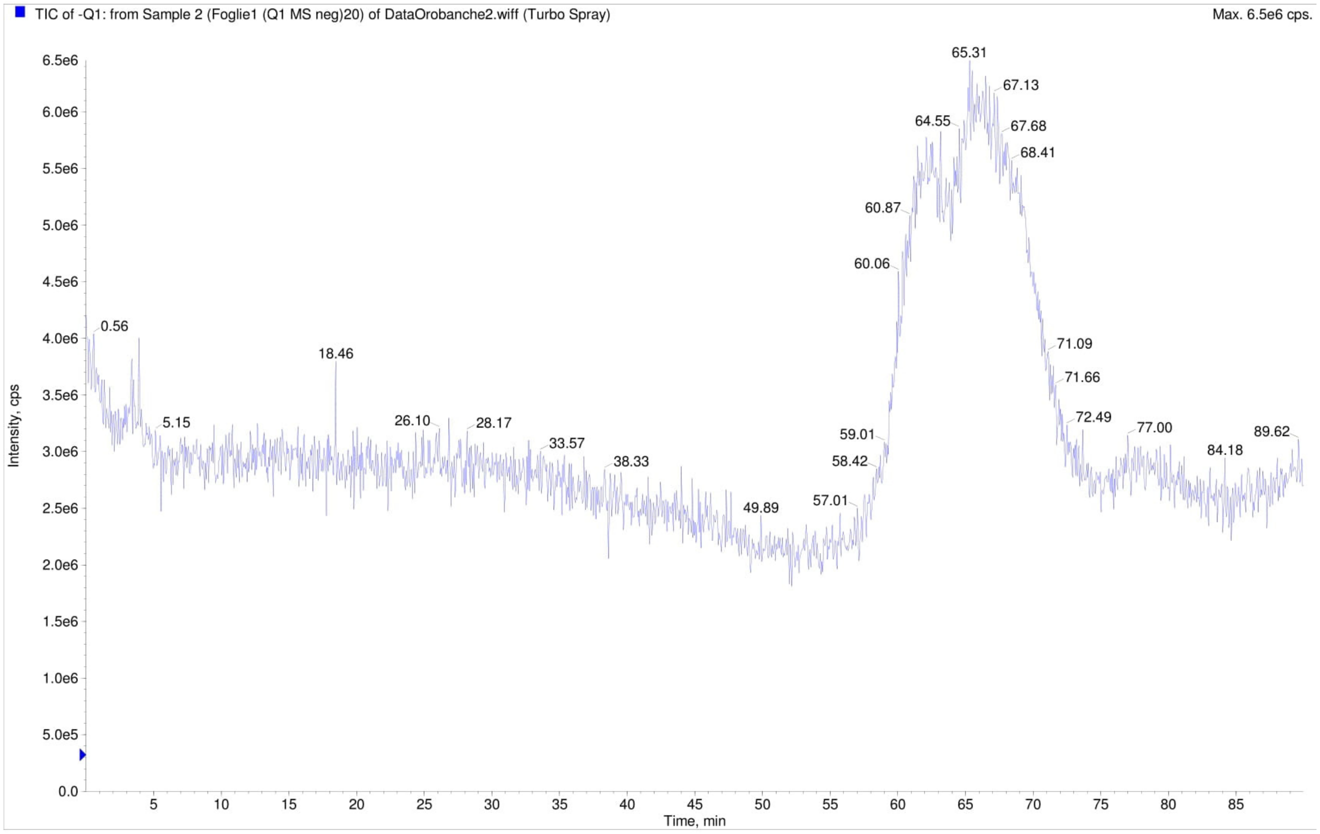

Figure S8. Mass spectrum of *Orobanche crenata* leaf extract (negative polarity – Q1 mode). In x-axis is reported the retention time (min).

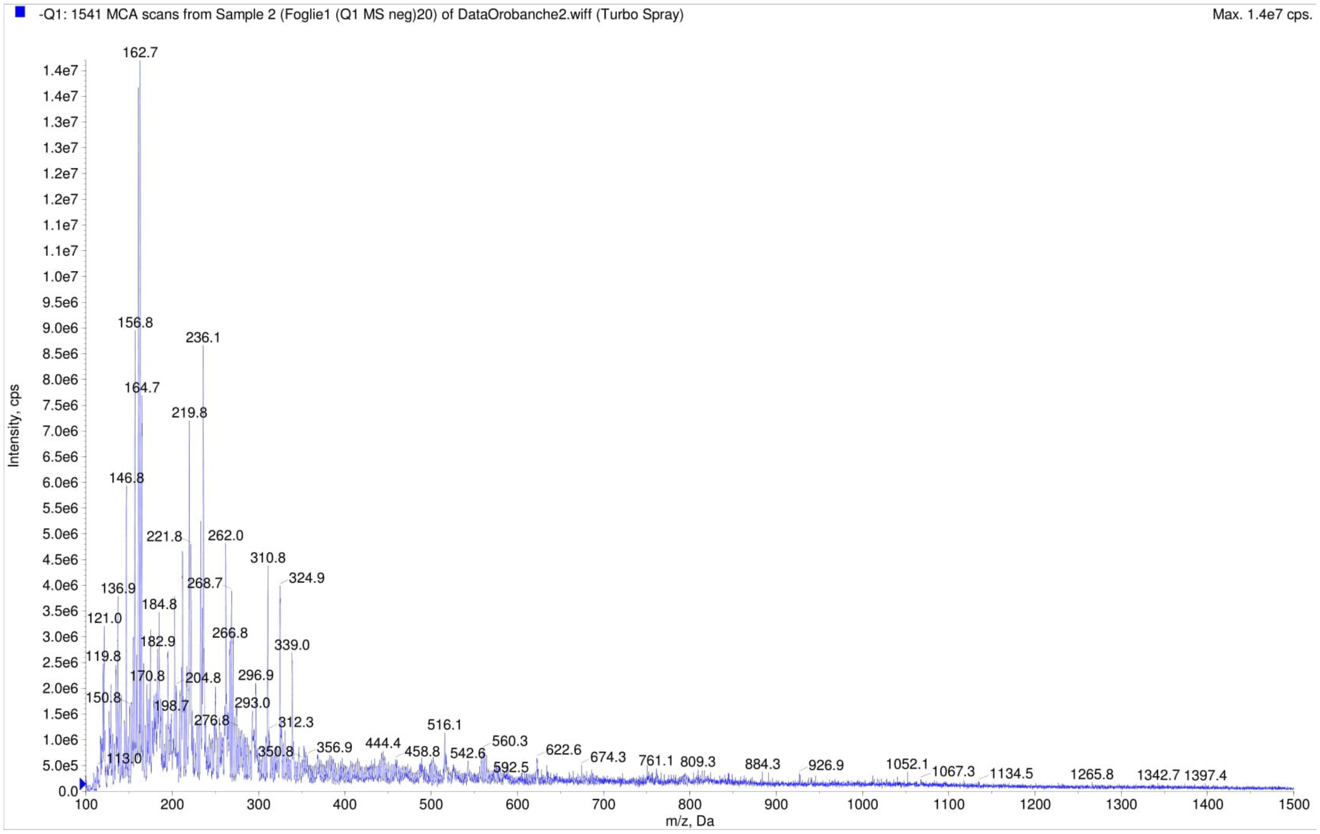

Figure S9. Mass spectrum of *Orobanche crenata* leaf extract (negative polarity – Q1 mode). In x-axis is reported the mass-to-charge ratio (m/z) from 100 to 1500 Da.

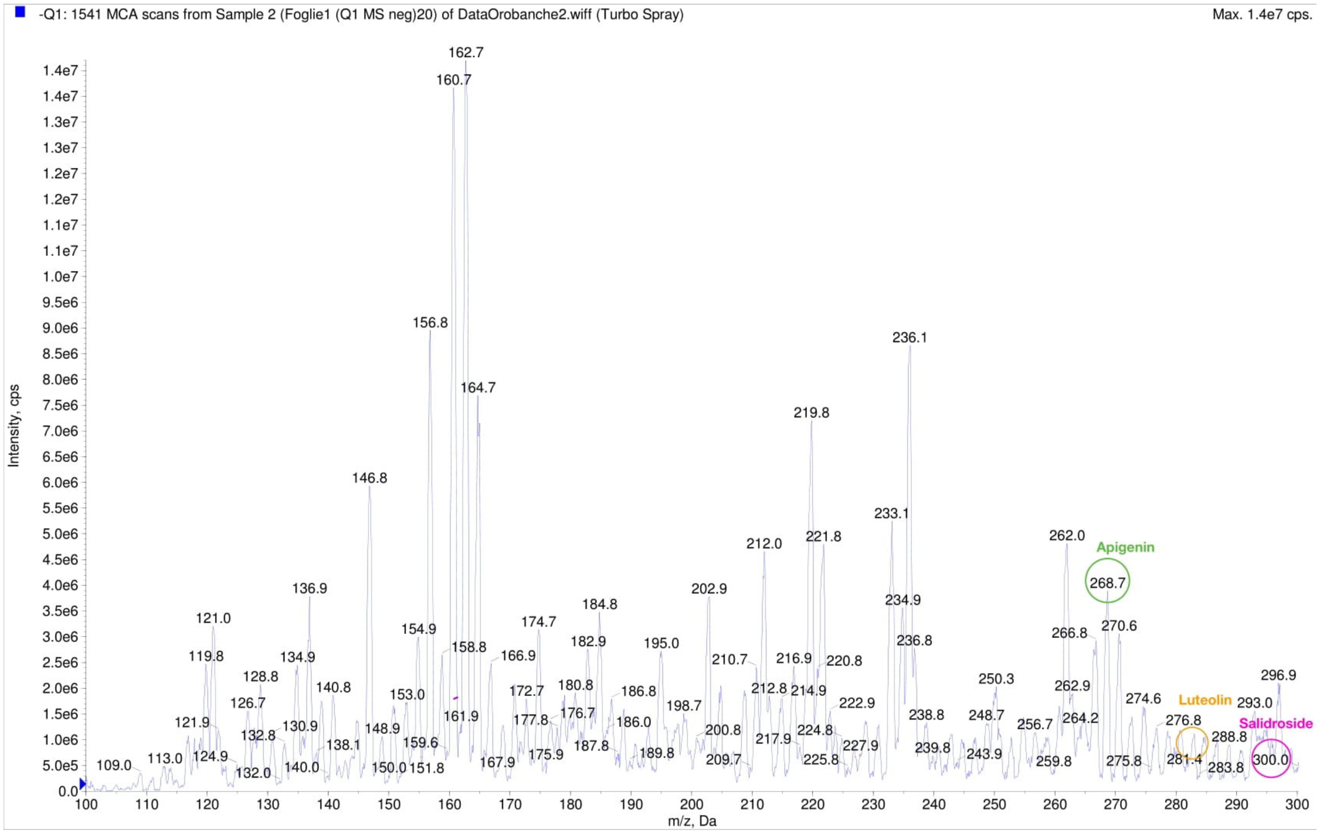

Figure S10. Mass spectrum of *Orobanche crenata* leaf extract (negative polarity – Q1 mode). In x-axis is reported the mass-to-charge ratio (m/z) from 100 to 300 Da.

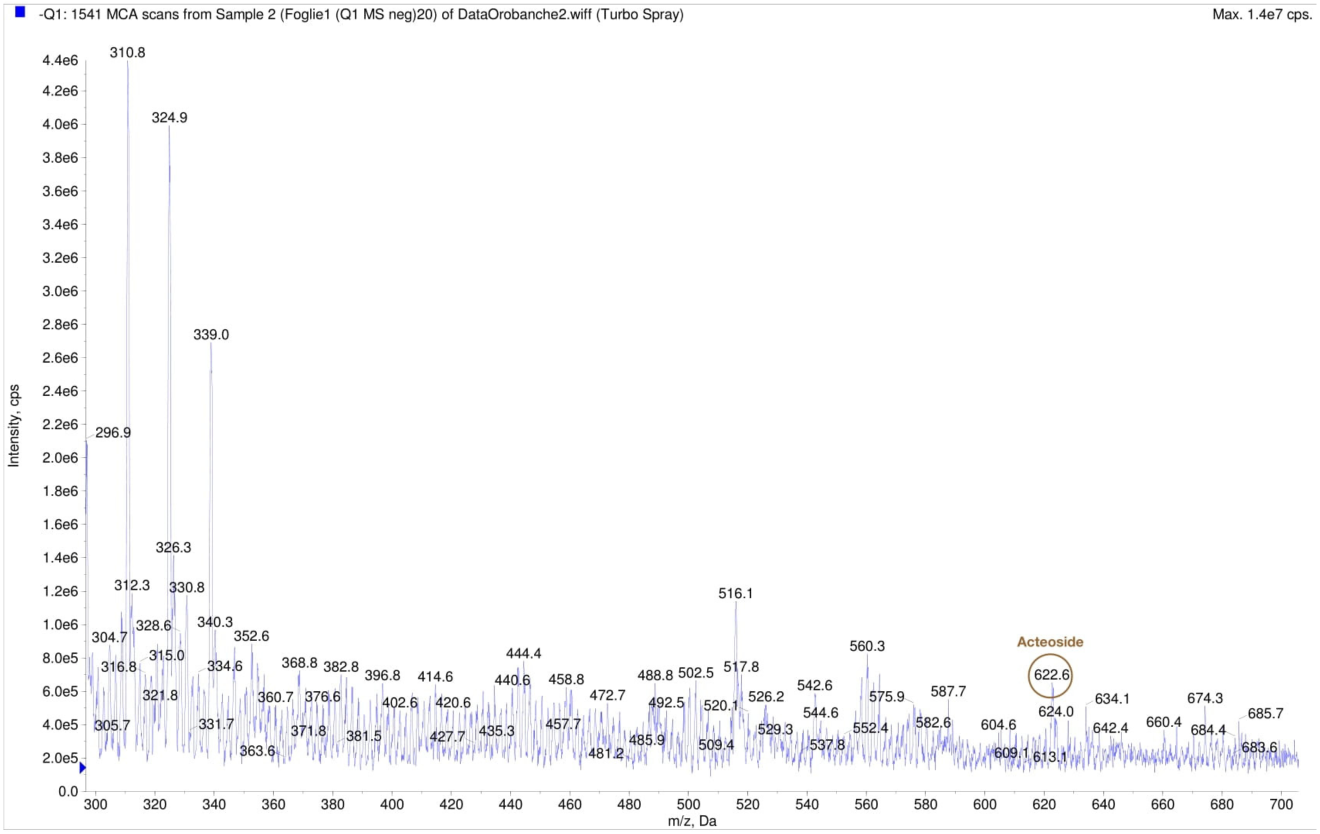

Figure S11. Mass spectrum of *Orobanche crenata* leaf extract (negative polarity – Q1 mode). In x-axis is reported the mass-to-charge ratio (m/z) from 300 to 700 Da.

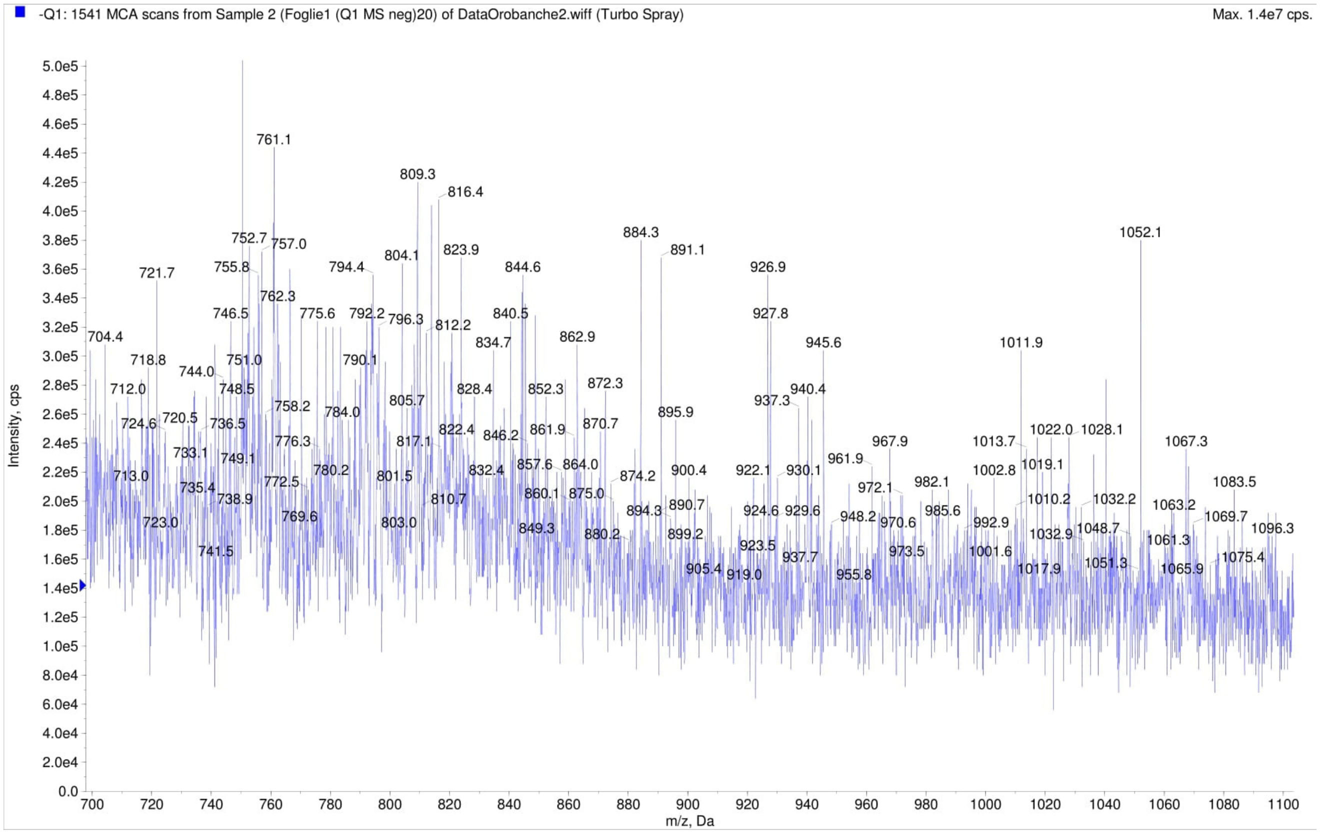

Figure S12. Mass spectrum of *Orobanche crenata* leaf extract (negative polarity – Q1 mode). In x-axis is reported the mass-to-charge ratio (m/z) from 700 to 1100 Da.

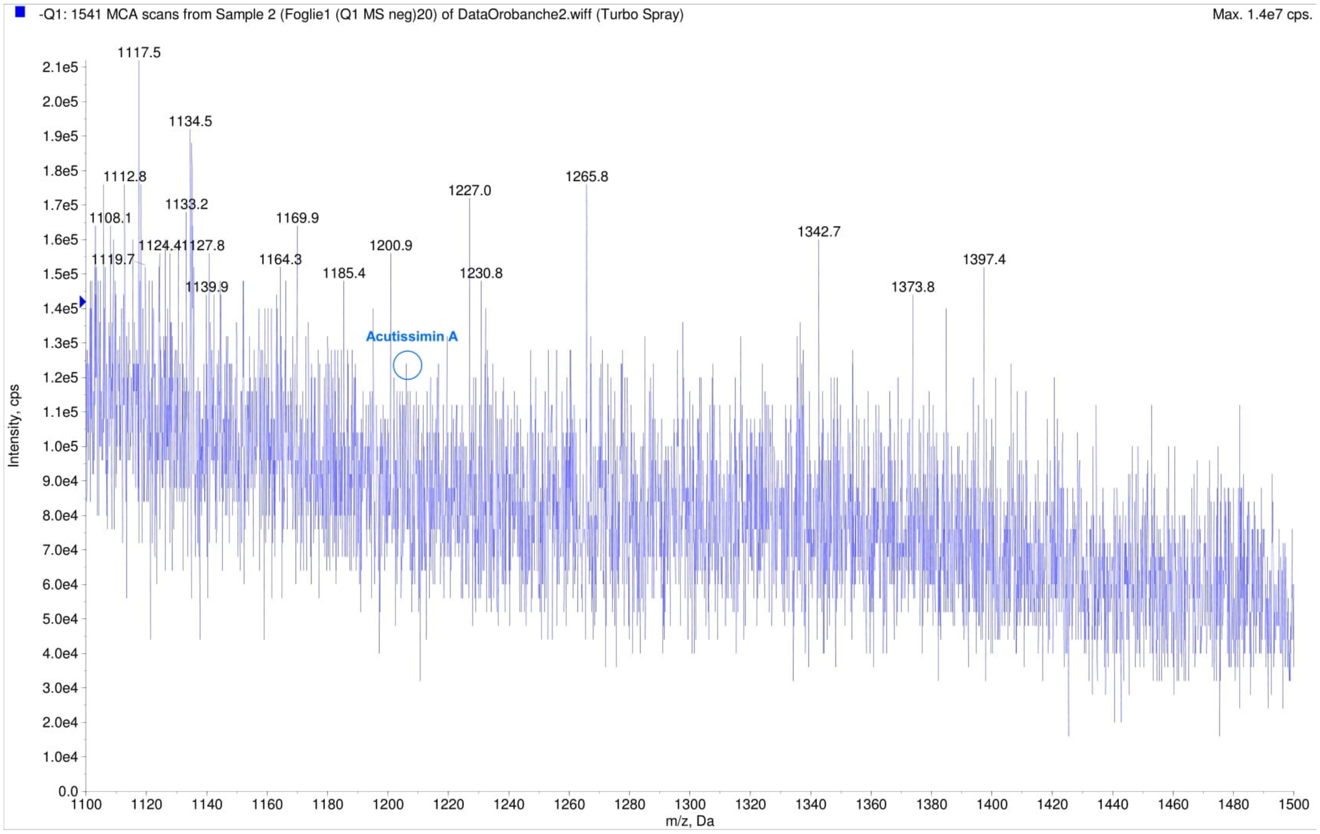

Figure S13. Mass spectrum of *Orobanche crenata* leaf extract (negative polarity – Q1 mode). In x-axis is reported the mass-to-charge ratio (m/z) from 1100 to 1500 Da.
